# Has intravenous lidocaine improved the outcome in horses following surgical management of small intestinal lesions in a UK hospital population?

**DOI:** 10.1186/s12917-016-0784-7

**Published:** 2016-07-27

**Authors:** Shebl E. Salem, Chris J. Proudman, Debra C. Archer

**Affiliations:** 1 Institute of Infection and Global Health, Department of Epidemiology and Population Health, University of Liverpool, Wirral, CH64 7TE UK; 2 Department of Surgery, Faculty of Veterinary Medicine, Zagazig University, Zagazig, Egypt; 3 School of Veterinary Medicine, University of Surrey, Guildford, GU2 7TE UK; 4 Philip Leverhulme Equine Hospital, School of Veterinary Science, University of Liverpool, Wirral, CH64 7TE UK

**Keywords:** Horse, Colic, Small intestine, Laparotomy, Lidocaine, Postoperative reflux, Postoperative ileus

## Abstract

**Background:**

Perioperative lidocaine treatment is commonly used in horses that undergo surgical treatment of colic, to prevent or treat postoperative ileus and reduce the effects of intestinal ischaemia-reperfusion injury. However, its clinical efficacy has not been evaluated in a large population of horses undergoing small intestinal surgery. The aim of the current study was to evaluate whether systemic lidocaine administration reduced the prevalence, volume and duration of postoperative reflux and improved rates of survival following surgical treatment of small intestinal lesions. Data were collected as a part of two prospective studies investigating postoperative survival of surgical colic patients admitted to a UK equine referral hospital during the periods 2004–2006 and 2012–2014. Kaplan-Meier plots of cumulative probability of survival and the log-rank test were used to compare survival between horses that did or did not receive lidocaine. The Wilcoxon rank-sum test was used to compare the total reflux volume and duration of reflux between the groups. A multivariable Cox proportional hazards model was used to identify pre- and intraoperative risk factors for non-survival.

**Results:**

Data from 318 horses were included in the final analysis. The overall prevalence of postoperative reflux was 24.5 %. This was significantly higher (34.8 %) in horses admitted in 2012–2014 compared to the 2004–2006 cohort (16.7). Perioperative lidocaine treatment had no effect on total reflux volume, duration of reflux or rates of postoperative survival nor was it a risk factor associated with altered postoperative survival. Variables identified to be associated with increased risk of postoperative mortality included packed cell volume on admission (hazard ratio [HR] 1.03 95 %, 95 % confidence interval [CI] 1.004–1.06, *p* = 0.024), heart rate on admission (HR 1.014, 95 % CI 1.004–1.024, *p =*0.008) and duration of surgery (HR 1.007, 95 % CI 1.002–1.01, *p* = 0.008).

**Conclusions:**

Lidocaine therapy had no effect on the prevalence of postoperative reflux, total reflux volume and duration of reflux nor did it have any effect on postoperative survival in horses undergoing surgical management of small intestinal disease for treatment of colic. There is a need for a well-designed multicentre, prospective randomised controlled trial to fully investigate the efficacy of lidocaine across different hospital populations.

**Electronic supplementary material:**

The online version of this article (doi:10.1186/s12917-016-0784-7) contains supplementary material, which is available to authorized users.

## Background

Postoperative ileus (POI) is a frequent complication in horses that have undergone small intestinal surgery for management of colic and it increases the risk of postoperative mortality [1–4]. In horses at high risk of POI or where POI develops postoperatively, prokinetic drugs are frequently used. Lidocaine, erythromycin, metoclopramide, cisapride, mosapride, and bethanechol are agents that have been evaluated as prokinetic drugs in horses through clinical [5–11] or experimental (both in vitro and in vivo) studies [12–16].

Lidocaine is a local anaesthetic agent, which has antiarrhythmic, analgesic and prokinetic properties when administered systemically in humans [17–19]. Several studies have also evaluated its effects in horses using in vitro and in vivo models. Systemically administered lidocaine has been found to have anti-inflammatory properties such as reduction of mucosal COX-2 expression and neutrophil count in ischaemic-injured equine intestine or amelioration of the negative effects of flunixin meglumine on recovery of injured mucosa [14, 15]. Lidocaine therapy has been proposed as a treatment option for horses with inflammatory conditions of the gastrointestinal tract including POI and recovery from ischaemic injury [20] and the authors of the latter study highlighted the need for further trials to evaluate its clinical effectiveness.

Studies that have investigated the prokinetic properties of lidocaine have reported conflicting results. A study that compared the effects of mosapride, cisapride, metoclopramide and lidocaine on jejunal motility in adult Thoroughbred horses found improved jejunal motility in response to cisapride, mosapride and metoclopramide but not with lidocaine treatment [16]. Additionally, lidocaine administration in normal horses that underwent laparotomy did not result in increased jejunal motility [13] and in a separate study was also associated with prolonged faecal transit time and reduced faecal output in normal horses [21]. More recent in vitro studies [22–24] reported positive effects of lidocaine on the contractility of smooth muscle isolated from equine jejunum in ischaemia and reperfusion injury models. The effect was found not to be specific to lidocaine and smooth muscle contractility was induced with other agents including mexiletine, bupivacaine, tetracaine and procaine [23]. Overall, limited evidence has been found to support the use of perioperative lidocaine as a therapy for POI [25]. Despite this, lidocaine therapy is frequently used in horses undergoing surgical treatment of colic. A recent survey of European Equine Internal Medicine and Surgery Diplomates reported that lidocaine was used intraoperatively in 50 % of cases and postoperatively in 67 % to prevent POI and was the drug of choice for 79 % of respondents in the management of POI in horses [26].

In the authors’ hospital, intravenous lidocaine has become more frequently used in the perioperative management of horses undergoing surgical management of small intestinal lesions for treatment of colic, and in particular horses at increased risk of POI. The aim of the current study was to investigate whether routine use of lidocaine in this population of horses has had any effect on the prevalence, duration of postoperative reflux and total reflux volume in horses, and to determine whether it has altered rates of postoperative survival or is a risk factor for altered postoperative survival.

## Methods

### Study population

The study population consisted of two cohorts of horses that underwent surgical treatment of colic related to a primary small intestinal lesion at a UK equine hospital. Horses were recruited onto the study if they underwent exploratory laparotomy for treatment of colic, were diagnosed with a small intestinal lesion at surgery, and stood in the recovery box following general anaesthesia. The first cohort were horses admitted in October 2004–November 2006, when lidocaine was only used intermittently in colic patients, and the second cohort was admitted in October 2012–November 2014 after lidocaine use had become implemented more routinely in horses undergoing small intestinal surgery for treatment of colic based on studies published between the two time periods advocating its use [3, 11, 20]. Routine surgical and postoperative management of colic patients during this time remained otherwise unchanged. Briefly, this consisted of intravenous flunixin meglumine administration for a minimum of 72 h (1.1 mg/kg bwt q. 12 h), and antimicrobial therapy with procaine penicillin (12 mg/kg bwt i.m. q. 12 h) or procaine penicillin and gentamicin (6.6 mg/kg bwt i.v. q. 24 h) for 3–5 days based on the surgical procedure performed (e.g. enterotomy, resection and anastomosis) and clinician preference. In this group of horses that had primary small intestinal lesions, all received intravenous fluid therapy (lactated Ringer’s solution) for at least the first 24 h postoperatively (unless they were able to tolerate oral fluids prior to this), and until oral fluid therapy could be initiated. Intravenous fluids were administered according to maintenance requirements (2 ml/kg bwt/h) together with additional fluid losses (e.g. net reflux volume) and were supplemented with calcium and potassium where required. Nasogastric intubation was performed at 2–4 hourly intervals once postoperative reflux developed. This continued until reflux ceased and oral water and feed were then gradually reintroduced over 3–4 days. Where reflux persisted, repeat laparotomy was undertaken or horses were euthanased based on lack of response to treatment and/or owner finances. Monitoring of small intestinal motility and diameter was performed ultrasonographically in some horses. Lidocaine therapy was usually administered during anaesthesia and immediately following placement in the stable following recovery from anaesthesia for most horses in the 2012–14 cohort unless clinician preference or lack of client finances dictated otherwise.

### Data collection and follow-up

Preoperative data were collected in a specifically designed colic admission form, which included questions about signalment, use, current management practices and any recent management changes, and results of clinical and clinicopathological examinations (Additional file 1). Postoperatively, clinical parameters including the net volume of reflux obtained and medications administered were recorded in specifically designed hospital forms. For both cohorts, survival was monitored following hospital discharge by telephone questionnaires administered with the horse owner/carer. This was performed every 3 months following discharge for the first 12 months and then at 6 monthly intervals. The questionnaires were administered as a part of two separate prospective studies investigating postoperative survival following colic surgery [27] and Salem et al. (unpublished data). All data were entered onto a Microsoft access or a Microsoft SQL Server database for the 2004–2006 and 2012–2014 cohorts respectively.

Administration of lidocaine intra- or postoperatively was recorded based on examination of the anaesthetic and postoperative monitoring sheets. The standard dose used at the hospital was a 1.3 mg/kg bolus given intravenously over 15 min followed by a 0.05 mg/kg bwt/min intravenous continuous rate infusion (CRI). Due to the fact that it is difficult to confirm true POI and to differentiate it from other causes of small intestinal obstruction e.g. mechanical obstruction that may occur postoperatively [28, 29], the term postoperative reflux (POR) [30, 31] was used in the current study. This was defined as a net nasogastric reflux of ≥2 l on at least two consecutive occasions within 24 h postoperatively [32]. Due to the fact that pain was not assessed using a single, consistent scoring system, we could not assess the effect of lidocaine on postoperative pain and this was not one of the aims of the current study.

### Data analysis

#### Descriptive data analysis

Survival time was calculated as a continuous variable starting from the date of surgery until the date of death or censoring. Horses were censored if they were lost to follow-up or were alive at the last interview date. Survival time was used to construct Kaplan-Meier plots of cumulative probability of survival and the log-rank test was used to compare survival between different surgical diagnosis categories, and between horses that did or did not receive lidocaine CRI intra- or postoperatively. A chi-square test of independence was used to compare frequencies of common surgical diagnosis categories and surgical procedures performed such as intestinal resection and anastomosis or types of anastomosis performed, use of prokinetic therapy and prevalence of POR between the two cohorts. The Wilcoxon rank-sum test was used to compare number of days that the horses were classified as having POR and total reflux volume between horses that received or did not receive lidocaine CRI intra- or postoperatively. Horses diagnosed with equine grass sickness (EGS) were excluded from statistical analyses [33].

#### Univariable analysis

Explanatory variables were screened for univariable association with time to death or censoring using univariable Cox proportional hazards models. Variables that had a likelihood ratio test (LRT) *p* value <0.25 were considered for inclusion into a multivariable model. Variables containing ≥30 % missing data were initially excluded from the analysis and categorical variables with few observations in some categories were re-categorised into fewer, biologically plausible categories. Pearson’s correlation coefficient was used to test for collinearity between explanatory variables. If variables were highly correlated (*r* ≥ 0.7) [34] or were considered to be measuring the same exposure, the variable that resulted in a greater reduction in residual deviance was selected [35]. The functional form of the relationships between continuous predictor variables and survival time was explored using penalized regression models [36]. The results from these models were examined graphically and variables that demonstrated a significant non-linear association with survival were fitted as P-spline smoothers, otherwise a linear fit was chosen. Data pre-processing and re-categorisation were performed using Epi Info7^1^ software. Survival analysis was performed using the ‘survival’ statistical package (version 2.38.1) [37] in R software environment version 3.2.2 [38]. Critical probability was set at 0.05 for all analyses.

#### Multivariable analysis

A multivariable Cox proportional hazards model considering only variables measured pre- and intraoperatively was built using a forward stepwise selection procedure. Variables were added sequentially into the model and were retained if they significantly improved model fit. This was indicated by a LRT *p* value <0.05 and a reduction in Akaike information criterion by at least 2 (ΔAIC >2) when nested models were compared. Eliminated variables were then forced back into the final model to assess for confounding and two-way interactions between variables remaining in this model were evaluated for statistical significance. The proportional hazard (PH) assumption was evaluated by plotting complementary log-log survival curves and scaled Schoenfeld residuals for variables remaining in the model. This was also assessed statistically using the Therneau-Grambsch non-proportionality test [39] as implemented by the ‘survival::cox.zph’ function in R. Scaled changes in regression coefficients associated with the exclusion of individual data points (delta-betas) were plotted in order to identify influential observations. The model was re-run following removal of any influential data point (−0.4 < delta-betas > 0.4) to evaluate their leverage on parameter estimates. Poorly fitted data points were also evaluated in a deviance residual plot. A data point was considered outlying if the corresponding deviance residual value is outside the range of (−2.5–2.5) [40]. The effect of surgeon and of anaesthetist on the probability of survival was tested in the final model by including each of these variables as a frailty term (random effect) in the final model.

## Results

Of 342 horses that underwent laparotomy for treatment of a primary small intestinal lesion, 318 were recruited onto the study (24 horses were excluded due to a diagnosis of EGS). The follow-up periods were the same for both study cohorts and all interviews were concluded approximately 3 months following the last recruitment date. This resulted in 272.6 horse years of recorded survival time. The number of horses in each study cohort and the frequencies of the most common surgical diagnosis categories and anastomoses types if small intestinal resection was performed are shown in Table 1. There was a significant increase in number of horses diagnosed with idiopathic focal eosinophilic enteritis (IFEE) lesions in the 2012–2014 cohort compared to the 2004–2006 cohort (19.7 vs 7.2 %). Surgical management of these lesions also varied: 78.6 % (11/14) of horses diagnosed with IFEE lesions in 2004–2006 admission years had intestinal resection and anastomosis during surgery compared with none in 2012–2014 admission years. There was a significant reduction in the frequency of horses in which intestinal resection and anastomosis was performed, particularly the frequency of side-to-side jejunocaecal anastomosis in recent admission years.

**Table 1:** Results of descriptive data analyses comparing the two study cohorts

Diagnosis/types of small intestinal anastomosis/prokinetic drugs/postoperative reflux	2004–2006 admission years	2012–2014 admission years	χ2 *p* value	Total
Horses diagnosed with small intestinal lesions	195 (60.6)	147 (55.7)	0.23	342 (58.4)
Common diagnosis categories				
Pedunculated lipoma obstruction	52 (25.7)	39 (26.5)	0.98	91 (26.6)
Idiopathic focal eosinophilic enteritis	14 (7.2)	29 (19.7)	0.001	43 (12.6)
Epiploic foramen entrapment	29 (14.9)	14 (9.52)	0.14	43 (12.6)
Ileal impaction	11(3.2)	13 (3.8)	0.7	24 (7)
Equine grass sickness (EGS)	15 (7.7)	9 (6.1)	0.6	24 (7)
Horses undergoing intestinal resection and anastomosis (EGS cases excluded)	115 (63.9)	56 (41.8)	<0.001	171 (54.5)
Side-to-side jejunocaecal anastomosis	42 (24.3)	12 (9)	0.001	54 (17.2)
Side-to-side ileocecal anastomosis	3 (1.7)	0 (0.0)	-	3 (0.96)
Side-to-side jejunojejunal anastomosis	4 (2.2)	0 (0.0)	-	4 (1.3)
End-to-end jejunoileal anastomosis	9 (5)	15 (11.2)	0.04	24 (7.6)
End-to-end jejunojejunal anastomosis	57 (31.7)	29 (21.6)	0.049	86 (27.4)
Prokinetic drugs used (EGS cases excluded)				
Lidocaine CRI intraoperatively	25 (16.1)	86 (62.3)	<0.001	111 (37.9)
Lidocaine CRI postoperatively	7 (4)	102 (73.9)	<0.001	109 (34.8)
Metoclopramide	1 (0.6)	9 (6.5)	-	10 (3.2)
Erythromycin	0 (0.0)	7 (5.07)	-	7 (2.2)
Lidocaine CRI plus Metoclopramide	0 (0.0)	8 (5.8)	-	8 (2.5)
Postoperative reflux (EGS cases excluded)	30 (16.7)	48 (34.8)	<0.001	78 (24.5)

Results of descriptive data analyses comparing frequencies of small intestinal lesions, common diagnosis categories, types of small intestinal anastomosis performed, prokinetic drugs administered intra- or postoperatively, and postoperative reflux between the two study cohorts. Chi-square (χ2) test *p* values comparing these frequencies and total frequencies are shown. Descriptive data are presented as numbers (%)

**Fig. 1:**
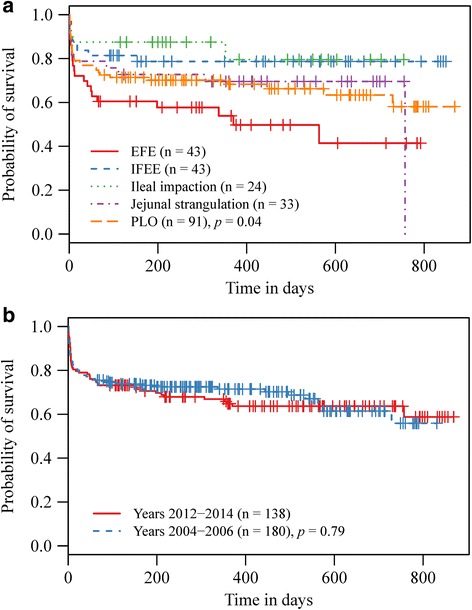
Kaplan-Meier plots comparing commonly identified small intestinal lesions (**a**) and the study cohorts (**b**). Kaplan-Meier plots of cumulative probability of survival and the log-rank test were used to compare survival between the five most frequent surgical diagnosis categories and between horses that belong to each of the admission periods (2004–2006 and 2012–2014 admission years). Vertical lines on the curves represent censoring times. The log-rank test *p* values and number of horses in each category are shown. EFE = epiploic foramen entrapment, IFEE = idiopathic focal eosinophilic enteritis, PLO = pedunculated lipoma obstruction

Cumulative probabilities of survival of horses in the five most frequent diagnosis categories are shown in Fig. 1a. IFEE and ileal impaction cases had a fairly stable survival at around 0.8 throughout the study period. This was in contrast to epiploic foramen entrapment (EFE) cases, which demonstrated a rapid decline in postoperative survival resulting in a median survival time of 370 days. The log-rank test of the probability of survival between these small intestinal lesions was significant at *p* = 0.038. Cumulative probability of survival did not differ significantly between horses in the two study cohorts (log-rank test *p* = 0.79) (Fig. 1b).

### Effect of lidocaine therapy

**Fig. 2:**
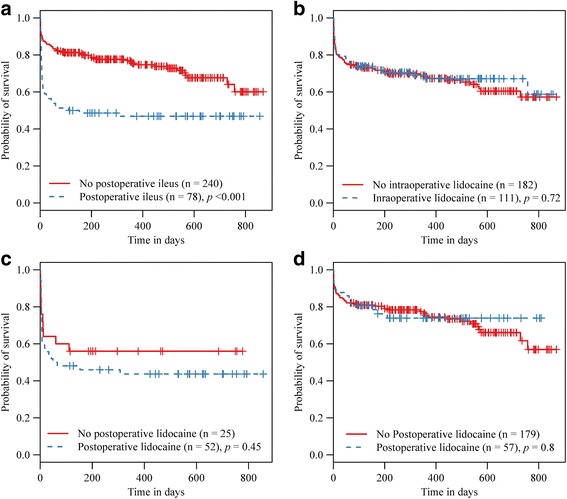
Kaplan-Meier plots illustrating the effect of POR and of lidocaine therapy on postoperative survival. Kaplan-Meier plots of cumulative probability of survival and the log-rank test were used to compare survival between horses that developed or did not develop postoperative reflux (**a**), horses that received or did not receive lidocaine CRI intraoperatively (**b**). Following stratifying the data by postoperative reflux, the effect of postoperative lidocaine therapy was assessed in horses that developed (**c**) or did not develop postoperative reflux (**d**). The number of horses in each category and the log-rank test *p* values are shown

**Fig. 3:**
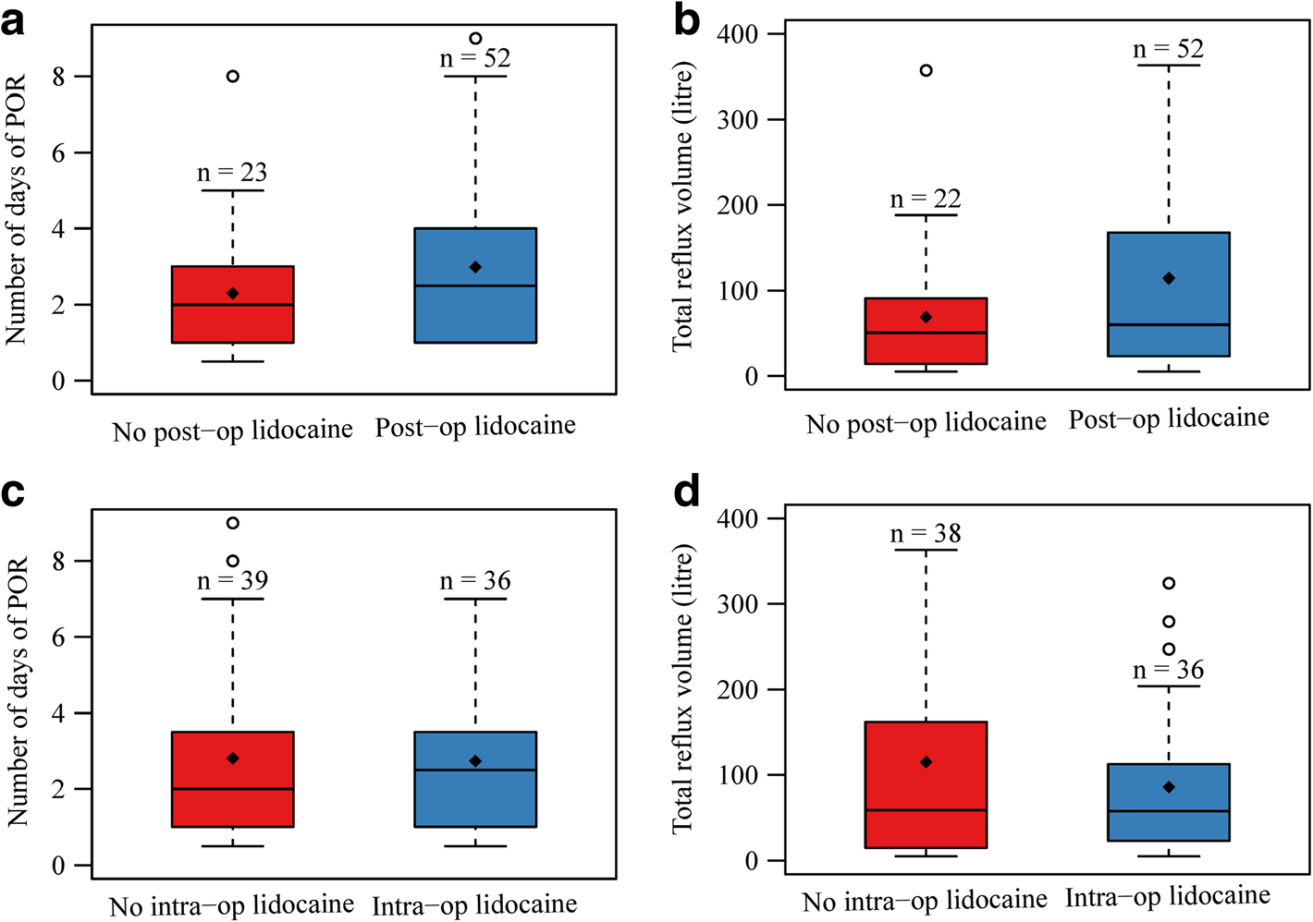
Boxplots demonstrating the effects of lidocaine therapy on duration of POR and total reflux volume. Duration of postoperative reflux (POR) and total reflux volume were compared between horses that were administered lidocaine continuous rate infusion postoperatively (**a** and **b**) or intraoperatively (**c** and **d**). The box represents the 25th and 75th percentiles of the data, the horizontal line across the middle of the box represents the median and the *dot* is the mean. Post-op = postoperatively, Intra-op = intraoperatively

The prevalence of POR in all horses recruited onto the study was 24.5 %. This was significantly higher in horses admitted in 2012–2014 (34.8 %) compared to 16.7 % in horses admitted in 2004–2006 (Table 1). POR resulted in a significant reduction in the long-term probability of survival and this effect was evident throughout the follow-up period (Fig. 2a). There was no statistical evidence that intraoperative lidocaine administration had any effect on long-term postoperative survival (log-rank test *p* = 0.72) (Fig. 2b). Because postoperative lidocaine treatment was not assigned randomly and horses were administered this medication if they were considered to be at high risk of developing POR or occasionally once horses had developed POR, the data were stratified and the effect of lidocaine CRI on probability of survival was compared in horses that did or did not develop POR separately. Such comparison did not reveal any significant improvement in the probability of survival in lidocaine-treated horses (Fig. 2c and d). The median duration of POR and of total reflux volume did not differ significantly between horses that were or were not administered intraoperative (Wilcoxon rank-sum *p* = 0.75 and 0.86 respectively) or postoperative lidocaine therapy (Wilcoxon rank-sum *p* = 0.15 and 0.14 respectively) (Fig. 3).

### Risk factors for postoperative survival

**Fig. 4:**
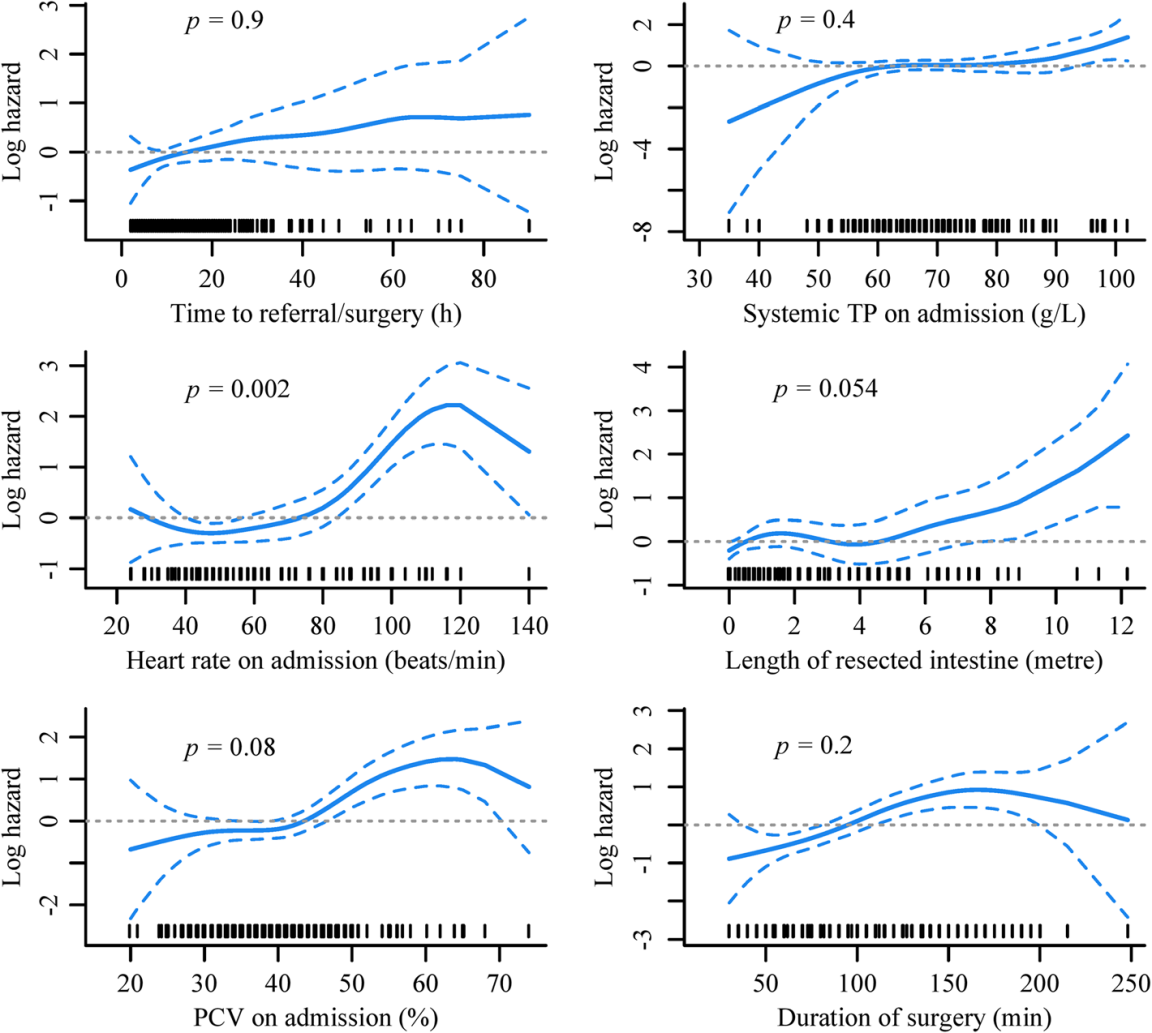
Plots of P-spline smoothers of continuous pre- and intraoperative variables. Penalised regression models were used to investigate the functional form of the relationships between the log hazard of postoperative death and continuous variables measured pre and intraoperatively. The plots show the fitted curves with 95 % confidence intervals (*dashed lines*) and the rug plots along the x-axes represent the number of data points. *Dotted horizontal lines* are at log hazard of zero. The *p* values are from chi square tests for non-linearity where a significant *p* value (*p* >0.05) indicates a non-linear association

Results of univariable analysis of continuous and categorical variables are available in Additional files 2 and 3. The functional form of the relationships examined by penalized regression models was non-linear for the variable heart rate on admission and therefore, this variable was evaluated as a penalized smoothed term in the multivariable model (Fig. 4). A final multivariable proportional hazards model is shown in Table 2. Variables identified to be associated with increased risk of postoperative mortality included packed cell volume on admission, heart rate on admission and duration of surgery. There was a significant multiplicative interaction between duration of surgery and EFE (hazard ratio 1.013, 95 % confidence interval 1.002–1.03, *p* = 0.027), meaning that the hazard ratio comparing horses with or without EFE depends on the duration of surgery. The random effects of surgeon and of anaesthetist tested in the final model were not significant (*p =* 0.98).

**Table 2:** Multivariable Cox proportional hazards model of variables associated with reduced likelihood of survival

Variable	Coefficient	Standard error	Hazard ratio	95 % CI of the hazard ratio	*p* value
Packed cell volume on admission (%)	0.032	0.014	1.033	1.004–1.06	0.024
Heart rate on admission (beats/min) “*the linear effect”*	0.014	0.005	1.014	1.004–1.024	0.008
Duration of surgery (min)	0.0066	0.0025	1.007	1.002–1.01	0.008
Epiploic foramen entrapment	Ref				
No	−0.66	0.77	0.52	0.1–1.35	0.39
Yes					
Surgery duration × EFE	0.013	0.006	1.013	1.002–1.03	0.027

The data are from 318 horses that survived following laparotomy for treatment of small intestinal lesions; only 302 horses were included in the model due to missing data for some variables. The model is adjusted for the non-linear relationship between heart rate on admission and probability of survival. *CI* confidence interval, *Re* reference category

The proportional hazards assumption was satisfied in the final multivariable model (global *p* value from the Therneau-Grambsch non-proportionality test = 0.94). A single influential data point was identified for variable PCV on admission (Additional file 4). The observations had a censored survival time, yet it was from a horse that had a PCV of 74 % on admission. Removal of this influential observation and re-running the model resulted in 33 % increase in the regression coefficient of this variable. The observation was found to be accurately recorded, so it was retained in the model. Four poorly fitting data points were identified on a deviance residual plot (Additional file 5). They had deviance residual values of >2.5 and the values were positive which meant observed death happened before the model was able to predict it. These observations were from horses that had PCV on admission of ≤40 % and surgery duration of ≤105 min, yet they had a survival time of 1 day.

## Discussion

The current study found no significant effect of intra- or postoperative lidocaine therapy on the duration of POR or total reflux volume in horses that underwent laparotomy for treatment of small intestinal lesions. Lidocaine administration also had no significant effect on postoperative survival, consistent with the findings of *Malone* et al. [11]. Lidocaine is currently commonly administered postoperatively in horses that have undergone surgical management of colic based on its perceived prokinetic properties [26]. These findings are important and demonstrate the need for further evaluation of the efficacy of lidocaine treatment in horses undergoing surgical management of colic related to small intestinal disease, particularly in horses at high risk of or that have developed POR, in a randomised controlled trial.

The prevalence of POR in the current study is comparable to previously reported rates of 9–33 % [3, 41–43]. The frequency of intra- or postoperative lidocaine treatment was significantly greater in horses that underwent surgical treatment of small intestinal lesions in 2012–2014 cohort, but the prevalence of POR doubled compared with the 2004–2006 admission years (34.8 vs 16.7 %), which was unexpected. The frequency of horses that were recovered from surgery that had undergone intestinal resection and anastomosis, particularly side-to-side jejunocaecal anastomoses was also significantly reduced in the 2012–2014 admission group. This is likely to be due to awareness of the effects of intestinal resection and anastomosis and of side-to-side jejunocaecal anastomosis on reduced postoperative survival, leading to some owners choosing to have horses euthanased on the operating table rather than continue with treatment or in equivocal cases, surgeons choosing not to resect potentially viable small intestine [27, 44–46]. It is not possible to determine why the prevalence of POR was increased in the 2012–2014 surgical cohort who routinely received perioperative lidocaine therapy, nor to attribute it directly to lidocaine use as this was not a randomised study. However, surgeons being more likely to leave small intestine that would undergo ischaemia-reperfusion injury may have resulted in increased prevalence of POR in the 2012–2014 cohort. The prevalence of IFEE lesions did differ between the two study periods which could have resulted in bias. Further exploration of the data using univariable and multivariable models with POR as the outcome demonstrated that the variable IFEE was not significantly associated with POR (*p* = 0.58) and inclusion of IFEE cases were therefore considered unlikely to have a significant impact on the study. In addition, whether resection was performed or not in IFEE cases was considered unlikely to alter the likelihood of postoperative mechanical obstruction occurring (and subsequent development of POR) as these are thickened lesions that cause acute colic due to simple obstruction at the site, and any physical thickening of intestine at the site of IFEE lesions left in situ (i.e. not resected) is comparable to that of an anastomosis.

In humans, systematic reviews and meta-analysis of randomised clinical trials have investigated the prokinetic and analgesic properties of lidocaine. Following open abdominal or laparoscopic surgery in adult humans, lidocaine has been found to be associated with significant reductions in postoperative pain scores [17, 47, 48]. However, the evidence for prokinetic efficacy is variable. Evidence of reduced time to first flatus, time to first bowel movement or likelihood of POI was found to be of low quality [17]. Comparison of the efficacy of 15 systemically acting prokinetic drugs for treatment of POI versus placebo in another systematic review reported that only lidocaine and neostigmine have an effect on gastrointestinal recovery in the form of reduction of time to first flatus and to first bowel movement [19]. Lidocaine treatment was also reported to be associated with reduced duration of hospital stay and of ileus following abdominal surgery in another systematic review [49].

The prokinetic properties of lidocaine have been evaluated previously in equine surgical colic patients in two separate randomised clinical trials [10, 11]. In one of these trials most of the horses were diagnosed with large intestinal lesions at surgery (20/28) and only two horses developed POI (one control, one treatment), and therefore the prokinetic properties of lidocaine could not be assessed [10]. In the study by *Malone* et al. [11], lidocaine administration to horses that developed nasogastric reflux resulted in significant reduction in number of horses refluxing at 30 h post-treatment and in duration of hospital stay compared to saline group. The study, however, did not report any differences in survival to hospital discharge or in the level of postoperative pain between groups. Limitations of the latter study include the fact that the study was performed on a small number of horses and included horses diagnosed with proximal duodenitis-jejunitis (DPJ) treated medically (8 DPJ and 24 POI horses), which are lesions with different pathobiology and relevance when considering lidocaine use in horses that have undergone small intestinal surgery [25]. A prophylactic effect of lidocaine in horses with POI was reported in a study by *Torfs* et al. [3]. In the latter study, lidocaine was frequently administered concurrently with other prokinetic drugs such as metoclopramide and this was not taken into account in the analysis of the data. Therefore, the evidence for the use of perioperative lidocaine to improve intestinal motility in horses at high risk of developing POI remains of low quality [25] and supports the need for further investigation.

We acknowledge that one of the key limitations to this study is the fact that horses were not randomly assigned to lidocaine treatment. Lidocaine was instead assessed as an intervention, as this was the only major change in management of horses undergoing surgical treatment of small intestinal lesions causing colic in the study hospital between the two study periods. Other factors that may have differed between the two time periods and may have had a potential effect include the effect of surgeon and anaesthetist, but they were assessed in the model and were not significantly associated with outcome. It is also important to note that economic factors may influence the decision to perform surgery or to recover horses following general anaesthesia [50, 51] and consequently this may introduce bias into survival models. The costs of treatment, including lidocaine therapy, may have been a limiting factor for some owners, particularly when postoperative reflux persisted for a number of days with no evidence of clinical improvement. It is, therefore, possible that with longer duration of therapy that some of these horses may have survived. We did not specifically investigate whether insurance cover had an effect on the surgical outcome. However, we have previously found this to have no effect on survival of horses recovered following surgery (unpublished hospital data) and the estimated costs of treatment are the same regardless of whether the horse is insured or not. It is also important to note that the effect of lidocaine on postoperative pain could not be assessed due to lack of consistent scoring in pain. This was not one of the key aims of the present study, but the analgesic effects of lidocaine should also be taken into consideration in future prospective trials evaluating its use in horses following intestinal surgery for colic.

In the current study, horses that underwent surgery for treatment of ileal impaction had the greatest probability of survival which is consistent with previous studies [43]. IFEE lesions were also associated with high rates of postoperative survival, with a relatively stable probability of survival at around 0.8 throughout the follow-up period. In contrast, EFE had a poor prognosis for survival with a median survival time of 370 days, which is again consistent with previous studies [4, 33, 43, 52]. In the current study, PCV and heart rate measured on admission and prolonged surgery duration were significantly and positively associated with postoperative mortality. PCV and heart rate can be markers for the degree of dehydration and systemic inflammatory response syndrome (SIRS)/endotoxaemia on admission [35]. This emphasises the importance of early diagnosis and treatment of equine surgical colic patients before development of marked physiological derangements/SIRS in order to maximise the probability of survival following laparotomy.

## Conclusions

In the current study, we found no effect of lidocaine therapy on frequency of POR, duration of reflux, nor was it a risk factor for, or associated with altered rates of postoperative survival. Despite the frequent use of lidocaine as a prokinetic agent in horses that develop or are at high risk of POI, the evidence for this still remains limited. Lidocaine therapy can add significantly to treatment costs making it important to fully evaluate the efficacy and cost-benefits of this drug. There is a need for a prospective, multicentre randomised clinical trial of lidocaine in horses with small intestinal lesions that has sufficient statistical power with well-defined inclusion criteria. There should also be a well-defined set of outcome measures including accurate measurement of POR duration and total reflux volume and use of objective and validated measures of postoperative pain, postoperative survival and complications.

## Abbreviations

CRI, continuous rate infusion; EFE, epiploic foramen entrapment; EGS, equine grass sickness; HR, hazard ratio; IFEE, idiopathic focal eosinophilic enteritis; intra-op, intraoperatively; PCV, packed cell volume; PLO, pedunculated lipoma obstruction; POI, postoperative ileus; POR, postoperative reflux; post-op, postoperatively

## Additional files

Additional file 1: The form used for initial data collection on hospital admission of each of the horses. (PDF 471 kb) Additional file 2: Continuous variables investigated for association with the risk of postoperative death. Data were collected from 318 horses that survived following general anaesthesia for treatment of small intestinal lesions and investigated for association with the risk of postoperative death using univariable Cox proportional hazards model. Descriptive data are presented as median (interquartile range). (DOCX 17 kb) Additional file 3: Categorical variables investigated for association with the risk of postoperative death. Data were collected from 318 horses that survived following general anaesthesia for treatment of small intestinal lesions and were investigated for association with the risk of postoperative death using univariable Cox proportional hazards model. (DOCX 22 kb) Additional file 4: Delta-betas plot of variables retained in the final Cox proportional hazards model. A single influential data point was evident for the variable packed cell volume (PCV) on admission. (PDF 38 kb) Additional file 5: Complementary log-log Kaplan-Meier survival curve for epiploic foramen entrapment (EFE) and a deviance residual plot. The complementary log-log Kaplan-Meier survival curve of EFE on a logarithmic scale (a) suggests that the proportional hazards assumption is satisfied for this variable (Therneau-Grambsch non-proportionality test *p* = 0.24). A deviance residual plot from the final multivariable Cox proportional hazards model (b) demonstrates five outlying data points (deviance residuals >2.5). (PDF 60 kb)

## Footnotes

1 Centre for Disease Control (CDC), Atlanta, Georgia, USA
