# Supplementary material for: Has intravenous lidocaine improved the outcome in horses following surgical management of small intestinal lesions in a UK hospital population?
Source: BMC Vet Res. 2016 Jul 27;12:157. doi: 10.1186/s12917-016-0784-7 (PMC4962447; doi:10.1186/s12917-016-0784-7)
Supplement: Additional file 1: — The form used for initial data collection on hospital admission of each of the horses. (PDF 471 kb) [file 12917_2016_784_MOESM1_ESM.pdf]

Colic Admission Form

CASE LABEL HERE

**Admission Date**	
**Admission Time**	

Please circle answers as appropriate. If an answer is unknown, please leave the field blank.

Premises Details

**Yard Name**		**Post Code**	
**Address**			

Signalment

**Gender**	Gelding / Mare / Stallion	**Pregnant?**	Y / N	**Days of Gestation**		days
If female:		**Nursing?**	Y / N	**Age of Foal**		months

**Breed**	TB / TBX / WB / Cob / Welsh / Shetland / Miniature / Draft / Arab / ID / IDX / Other:	

**Age**		years	**Weight**		Kg	**Height**		hh

**How long has the horse been in the owner's possession?**		years		months

General History

**Type of Work**	General Riding / Racing / Eventing / SJ / Breeding / Dressage / Retired / Other:		
**Level of Work**		hours/week	**Intensity of Work**	None / Light / Medium / Intense

**Management**	Private Stable / Livery Yard / Racing Yard / Professional Yard / Other:			
**Currently Stabled?**	Y / N	**Hours Stabled Per Day**		hours

**Main Carer**	Owner / Private Groom / Professional Yard Staff / Loaner / Other:	
**Number of Carers During Last 2 Weeks**		

**Other Horses at the Property?**	Y / N	**If so, how many?**	

**Stereotypic Behaviour**	None / Wind Sucking / Weaving / Box Walking / Crib Biting

**Bedding**	Shavings / Straw / Paper / At grass / Pellets / Hemp / Other:	

**Forage**	Hay / Soaked Hay / Haylage / Grass / Silage Other:	
**Forage Frequency**		times per day
**Hard Feed**	None / Coarse Mix / Pellets/Cubes / Oats / Bran / Barley / Sugar Beet / Corn / Carrot / Mash / Alfa Alfa / Apple / Grass nuts / Chaff / Other:	
**Hard Feed Frequency**		times per day

**Supplements Given?**	Y / N
**Supplements Details**	

Worming History

**Worming Strategy**	Every 6-12 weeks / Every 13 weeks - 6 months / Every 6-12 months / Targeted	
**Weeks Since Last Worming**		weeks
**Last Worming Product Used**		
**Faecal Egg Count Performed in Last 6 Months?**	Y / N	
If yes: **Faecal Egg Count Result**	Normal / Abnormal	
**Tapeworm ELISA Performed in Last 6 Months?**	Y / N	
If yes: **Tapeworm ELISA Result**	Normal / Abnormal	

Please circle answers as appropriate. If an answer is unknown, please leave the field blank.

**Dental Care**		
**Is There a Dental Care Routine?**	Y / N	
**Dentist**	Vet / Dental Technician / Other:	
**Dental Care Frequency**	6 monthly / 12 monthly / More than 12 monthly	
**Light Source Used?**	Y / N	
**Mouth Gag Used?**	Y / N	
**Known Dental Problems?**	Y / N	
If yes: **Please Give Details**		

**Changes In The Past 30 Days**	
**Changes In Feeding?**	Y / N
If yes: **Type of Change**	Hard feed introduction / Change hard feed type / Change forage type / Change forage source / Supplement introduction / Change supplements / Other:

**Changes In Stabling/Turnout?**	Y / N
If yes: **Type of Change**	Decrease in turn-out / Increase in turn-out / Change of pasture / Box rest / Other:

**Transportation?**	Y / N	
If yes: **Transportation Duration**		hours
If yes: **Days Since Transportation**		days

**Medical Problems Other Than Colic?**	Y / N	
If yes: **Other Medical Problems**	Diarrhoea / Fever / Laminitis / Respiratory / Lameness / Urinary infection / Other:	

**Medications Received?**	Y / N	
If yes: **What Medications Received**	Sedation / Anthelmintic / NSAID / Corticosteroids / Buscopan / Antibiotics / Other:	

**Other History**	
**Number of Previous Colic Episodes in Past 12 Months:**	
**Did a Vet Attend?**	Y / N

**Previous Colic Surgery?**	Y / N
If yes: **Details of Previous Colic Surgery (e.g. Date)**	
If yes: **Diagnosis**	

**Non-Abdominal Surgery in Past 6 Months?**	Y / N
If yes: **Details of Non-Abdominal Surgery (e.g. Date)**	

Please circle answers as appropriate. If an answer is unknown, please leave the field blank.

Current Colic History

**When was the horse last seen to be normal?**	Date		Time	
**When was the colic first observed?**	Date		Time	

**Signs of Colic**	Rolling / Muscle tremors / Trouble swallowing / Sweating / Teeth grinding / Flank watching / Pawing ground / Getting up and down / Kicking belly / Lying still / Crouching / Other:

Owner's Perception of Pain Severity (ask owner to mark a cross on the line below)

Pain free = 0 ———————————————— 10 = Severe pain

**Number of Vet Visits**		
**Drugs Administered**	Flunixin / Phenylbutazone / Other NSAIDS / Alpha 2 / Butorphanol / Mineral Oil / Buscopan / Other:	
**Response to Treatment**	None / Improved / Deteriorated	

Clinical Examination

**Attitude**	Normal / Painful / Quiet / Unwilling to move

Pain Severity (mark a cross on the line below)

Pain free = 0 ———————————————— 10 = Severe pain

**Body Condition Score**	1	2	3	4	5	6
	Very Poor	Poor	Moderate	Good	Fat	Very Fat

**Other Signs**	Salivation / Muscle fasciculations / Poor tail tone / Sweating / Other:	

**Cleanliness of the Horse**	Clean clipped / Clean unclipped / Moderate / Filthy

Cardiovascular System

**Heart Rate**		beats/min		**Pulse Quality**	Normal / Weak / Bounding

**Mucous Membranes**	Normal / Congested / Cyanotic / Pale / Jaundiced

**CRT**		secs.	**PCV**		%	**TP**		g/l	**Blood Lactate**		mmol/l

**Rectal Temperature**		˚C	**Respiratory Rate**		breaths/min

Please circle answers as appropriate. If an answer is unknown, please leave the field blank.

Gastrointestinal System

**Abdominal Distension?**	Y / N

**Ausculation**	Normal / Increased / Reduced / None

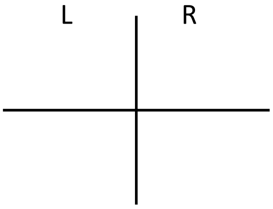

**Rectal Exam Findings**	Normal rectal findings	Distended small intestine	Gas distended large colon
	Large colon impaction	Small colon distension	Left dorsal displacement
	Right dorsal displacement	Caecal impaction	Pregnant/Enlarged uterus
	Gas distended viscus	Tight taenia band	Small intestine impaction
	Other:		

**Nasogastric Tube Passed?**	Y / N	
If yes: **Gastric Reflux?**	Y / N	
If yes: **Gastric Reflux Net Volume**		litres

**Paracentesis Performed?**	Y / N
If yes: **Gross Appearance**	Normal / Turbid / Sanguinous / Blood contaminated / Gut contents / Chylous / No fluid obtained / Other:

**Peritoneal WBC**		$X10^9$/litre	**Peritoneal TP**		g/litre	**Peritoneal Lactate**		mmol/l

**Abdominal Ultrasound Performed?**	Y / N
**Ultrasound Findings**	Amotile SI / Distended SI / Increased volume of fluid / Colon distension / Unremarkable / Other:

Initial Management

**Initial Management**	Surgical / Medical / Euthanased After Exam

**Provisional Diagnosis**	
