# Supplementary material for: Has intravenous lidocaine improved the outcome in horses following surgical management of small intestinal lesions in a UK hospital population?
Source: BMC Vet Res. 2016 Jul 27;12:157. doi: 10.1186/s12917-016-0784-7 (PMC4962447; doi:10.1186/s12917-016-0784-7)
Supplement: Additional file 2: — Continuous variables investigated for association with the risk of postoperative death. Data were collected from 318 horses that survived following general anaesthesia for treatment of small intestinal lesions and investigated for association with the risk of postoperative death using univariable Cox proportional hazards model. Descriptive data are presented as median (interquartile range). (DOCX 17 kb) [file 12917_2016_784_MOESM2_ESM.docx]

**Supplementary Table 1.**

Continuous variables investigated for association with the risk of postoperative death.

| Variable | Median (interquartile range) | Coefficient | Standard error | Hazard ratio | 95% confidence interval of the hazard ratio | LRT *p* value | % of missing data |
| --- | --- | --- | --- | --- | --- | --- | --- |
| Age (years) | 13 (9, 17) | 0.0002 | 0.016 | 1 | 0.99–1.03 | 0.99 | 0.9 |
| Weight (kg) | 508.5 (434.5, 578.5) | 0.001 | 0.001 | 1.001 | 0.99–1.002 | 0.51 | 1.9 |
| Time to referral (h) | 12.5 (8, 20.8) | 0.014 | 0.007 | 1.014 | 1.001–1.028 | 0.03 | 19.5 |
| Heart Rate (bpm) | 52 (44, 70) | 0.022 | 0.004 | 1.02 | 1.014–1.03 | <0.001 | 2.8 |
| Packed call volume (%) | 39 (34, 44) | 0.05 | 0.01 | 1.05 | 1.03–1.07 | <0.001 | 3.1 |
| Total Protein (g/l) | 68 (62, 75) | 0.025 | 0.01 | 1.025 | 1.006–1.045 | 0.01 | 4.4 |
| Resection length (metres) | 0.61 (0, 2.4) | 0.1 | 0.04 | 1.12 | 1.04–1.2 | 0.005 | 0.63 |
| Duration of surgery (min) | 90 (65, 125) | 0.01 | 0.002 | 1.01 | 1.007–1.015 | <0.001 | 0.63 |
| Heart rate at 12 h postoperatively (bpm) | 52 (44, 64) | 0.03 | 0.006 | 1.03 | 1.02–1.04 | <0.001 | 6.3 |
| Heart rate at 36 h postoperatively (bpm) | 44 (36, 48) | 0.036 | 0.05 | 1.04 | 1.03–1.05 | <0.001 | 10.4 |

Data were collected from 318 horses that survived following general anaesthesia for the treatment of small intestinal lesions and investigated for association with the risk of postoperative death using a univariable Cox proportional hazards model. Descriptive data are presented as median (interquartile range). LRT = likelihood ratio test, bpm = beats per minute
