# Supplementary material for: Has intravenous lidocaine improved the outcome in horses following surgical management of small intestinal lesions in a UK hospital population?
Source: BMC Vet Res. 2016 Jul 27;12:157. doi: 10.1186/s12917-016-0784-7 (PMC4962447; doi:10.1186/s12917-016-0784-7)
Supplement: Additional file 3: — Categorical variables investigated for association with the risk of postoperative death. Data were collected from 318 horses that survived following general anaesthesia for treatment of small intestinal lesions and were investigated for association with the risk of postoperative death using univariable Cox proportional hazards model. (DOCX 22 kb) [file 12917_2016_784_MOESM3_ESM.docx]

**Supplementary Table 2.**

Categorical variables investigated for association with the risk of postoperative death

| Variable | Category | Descriptive data  No. (%) | Coefficient | Standard error | Hazard ratio | 95% confidence interval of the hazard ratio | LRT *p* value | % of missing data |
| --- | --- | --- | --- | --- | --- | --- | --- | --- |
| Animal breed | TB / TBX | 86 (29.7) | Ref. |  |  |  |  | 9.12 |
|  | WB / WBX | 26 (9.0) | 0.24 | 0.36 | 1.27 | 0.63–2.57 |  |  |
|  | Draft / Cob | 71 (24.57) | 0.14 | 0.38 | 1.15 | 0.66–1.99 |  |  |
|  | Pony | 50 (17.30) | −0.15 | 0.33 | 0.86 | 0.45–1.65 |  |  |
|  | Other | 56 (19.38) | 0.036 | 0.3 | 1.04 | 0.58–1.87 | 0.88 |  |
| Sex | Female | 108 (34.0) | Ref. |  |  |  |  | 0.0 |
|  | Male | 210 (66.04) | 0.4 | 0.22 | 1.5 | 0.97–2.3 | 0.06 |  |
| Surgery performed out of hours | No | 111 (35.016) | Ref. |  |  |  |  | 0.31 |
|  | Yes | 206 (65.0) | −0.18 | 0.2 | 0.8 | 0.6–1.24 | 0.37 |  |
| Season of admission | Spring | 88 (27.67) | Ref. |  |  |  |  | 0.0 |
|  | Summer | 77 (24.21) | −0.05 | 0.27 | 0.95 | 0.55–1.63 |  |  |
|  | Autumn | 92 (28.93) | −0.12 | 0.26 | 0.88 | 0.53–1.5 |  |  |
|  | Winter | 61 (19.18) | −0.07 | 0.28 | 0.93 | 0.53–1.6 | 0.97 |  |
| Reflux on admission | No | 184 (75.41) | Ref. |  |  |  |  | 23.3 |
|  | Yes | 60 (24.60) | 0.44 | 0.24 | 1.55 | 0.97–2.5 | 0.07 |  |
| Guaiacol glycerine ether for anaesthesia induction | No | 288 (90.57) | Ref. |  |  |  |  | 0.0 |
|  | Yes | 30 (9.43) | −0.4 | 0.4 | 0.67 | 0.3–1.5 | 0.28 |  |
| Thiopentone for anaesthesia induction | No | 279 (87.74) | Ref. |  |  |  |  |  |
|  | Yes | 39 (12.26) | −0.026 | 0.3 | 0.97 | 0.53–1.78 | 0.93 |  |
| Ketamine-based anaesthesia induction | No | 34 (10.7) | Ref. |  |  |  |  |  |
|  | Yes | 284 (39.31) | 0.05 | 0.3 | 1.05 | 0.55–2.02 | 0.88 |  |
| Anaesthetic inhalation agent | Sevoflurane | 65 (21.52) | Ref. |  |  |  |  | 5.03 |
|  | Halothane | 11 (3.64) | −0.33 | 0.61 | 0.7 | 0.22–2.4 |  |  |
|  | Isoflurane | 226 (74.83) | −0.19 | 0.23 | 0.83 | 0.52–1.31 | 0.68 |  |
| Lidocaine during anaesthesia | No | 182 (62.12) | Ref. |  |  |  |  | 7.86 |
|  | Yes | 111 (37.88) | −0.08 | 0.2 | 0.92 | 0.6–1.4 | 0.72 |  |
| Small intestinal anastomosis | No | 147 (46.23) | Ref. |  |  |  |  | 0.0 |
|  | All side to side anastomoses | 61 (19.18) | 0.63 | 0.25 | 1.9 | 1.14–3.1 |  |  |
|  | All end to end anastomoses | 110 (34.6) | 0.47 | 0.23 | 1.6 | 1.02–2.5 | 0.02 |  |
| Side to side small intestine anastomosis | No | 257 (80.82) | Ref. |  |  |  |  | 0.0 |
|  | Yes | 61 (19.18) | 0.4 | 0.2 | 1.5 | 0.97–2.3 | 0.08 |  |
| Resection | No | 150 (47.17) | Ref. |  |  |  |  | 0.0 |
|  | Yes | 168 (52.83) | 0.6 | 0.2 | 1.77 | 1.2–2.6 | 0.004 |  |
| Pelvic flexure enterotomy | No | 267 (84.76) | Ref. |  |  |  |  | 0.94 |
|  | Yes | 48 (15.24) | 0.47 | 0.24 | 1.6 | 0.99–2.6 | 0.06 |  |
| Pedunculated lipoma obstruction | No | 227 (71.38) | Ref. |  |  |  |  | 0.0 |
|  | Yes | 91 (28.62) | 0.03 | 0.2 | 1.03 | 0.68–1.6 | 0.88 |  |
| Idiopathic focal eosinophilic enteritis | No | 275 (86.48) | Ref. |  |  |  |  | 0.0 |
|  | Yes | 43 (13.52) | −0.61 | 0.35 | 0.55 | 0.28–1.08 | 0.06 |  |
| Epiploic foramen entrapment | No | 275 (86.48) | Ref. |  |  |  |  |  |
|  | Yes | 43 (13.52) | 0.59 | 0.24 | 1.8 | 1.12–2.9 | 0.02 | 0.0 |
| Repeat laparotomy | No | 289 (90.88) | Ref. |  |  |  |  |  |
|  | Yes | 29 (9.12) | 1 | 0.25 | 2.7 | 1.65–4.5 | <0.001 |  |
| Postoperative reflux | No | 240 (75.47) | Ref. |  |  |  |  | 0.0 |
|  | Yes | 78 (24.52) | 0.87 | 0.2 | 2.4 | 1.62–3.6 | <0.001 |  |
| Postoperative colic | No | 168 (52.83) | Ref. |  |  |  |  | 0.0 |
|  | Yes | 150 (47.17) | 0.5 | 0.2 | 1.65 | 1.1–2.4 | 0.01 |  |
| Diarrhoea | No | 299 (94.03) | Ref. |  |  |  |  | 0.0 |
|  | Yes | 19 (5.97) | 0.014 | 0.4 | 1.014 | 0.47–2.18 | 0.97 |  |
| Purulent incisional discharge | No | 234 (73.58) | Ref. |  |  |  |  | 0.0 |
|  | Yes | 84 (26.42) | −0.052 | 0.24 | 0.56 | 0.37–0.96 | 0.026 |  |
| Postoperative lidocaine treatment | No | 204 (65.18) | Ref. |  |  |  |  | 1.6 |
|  | Yes | 109 (34.82) | 0.37 | 0.19 | 1.45 | 0.97–2.13 | 0.06 |  |
| Postoperative lidocaine plus metoclopramide | No | 305 (97.44) | Ref. |  |  |  |  | 1.6 |
|  | Yes | 8 (2.56) | 1.66 | 0.37 | 5.24 | 2.52–10.91 | <0.001 |  |
| Admission years | 2012–2014 | 138 (43.4) | Ref. |  |  |  |  | 0.0 |
|  | 2004–2006 | 180 (56.6) | −0.053 | 0.19 | 0.95 | 0.65–1.39 | 0.79 |  |

Data were collected from 318 horses that survived following general anaesthesia for the treatment of small intestinal lesions and investigated for association with the risk of postoperative death using a univariable Cox proportional hazards model. LRT = likelihood ratio test, TB/TBX = Thoroughbred/Thoroughbred cross, WB/WBX = Warmblood/Warmblood Cross, Ref. = reference category, Season of admission classified as spring (March – May), summer (June – August), autumn (September – November) and winter (December – February), Surgery performed out of hours was defined as surgery performed between the hours of 5pm – 9am Monday – Friday and at any time over the days of Saturday or Sunday. Descriptive data are presented as numbers and percentages
