# Supplementary material for: Has intravenous lidocaine improved the outcome in horses following surgical management of small intestinal lesions in a UK hospital population?
Source: BMC Vet Res. 2016 Jul 27;12:157. doi: 10.1186/s12917-016-0784-7 (PMC4962447; doi:10.1186/s12917-016-0784-7)
Supplement: Additional file 4: — Delta-betas plot of variables retained in the final Cox proportional hazards model. A single influential data point was evident for the variable packed cell volume (PCV) on admission. (PDF 38 kb) [file 12917_2016_784_MOESM4_ESM.pdf]

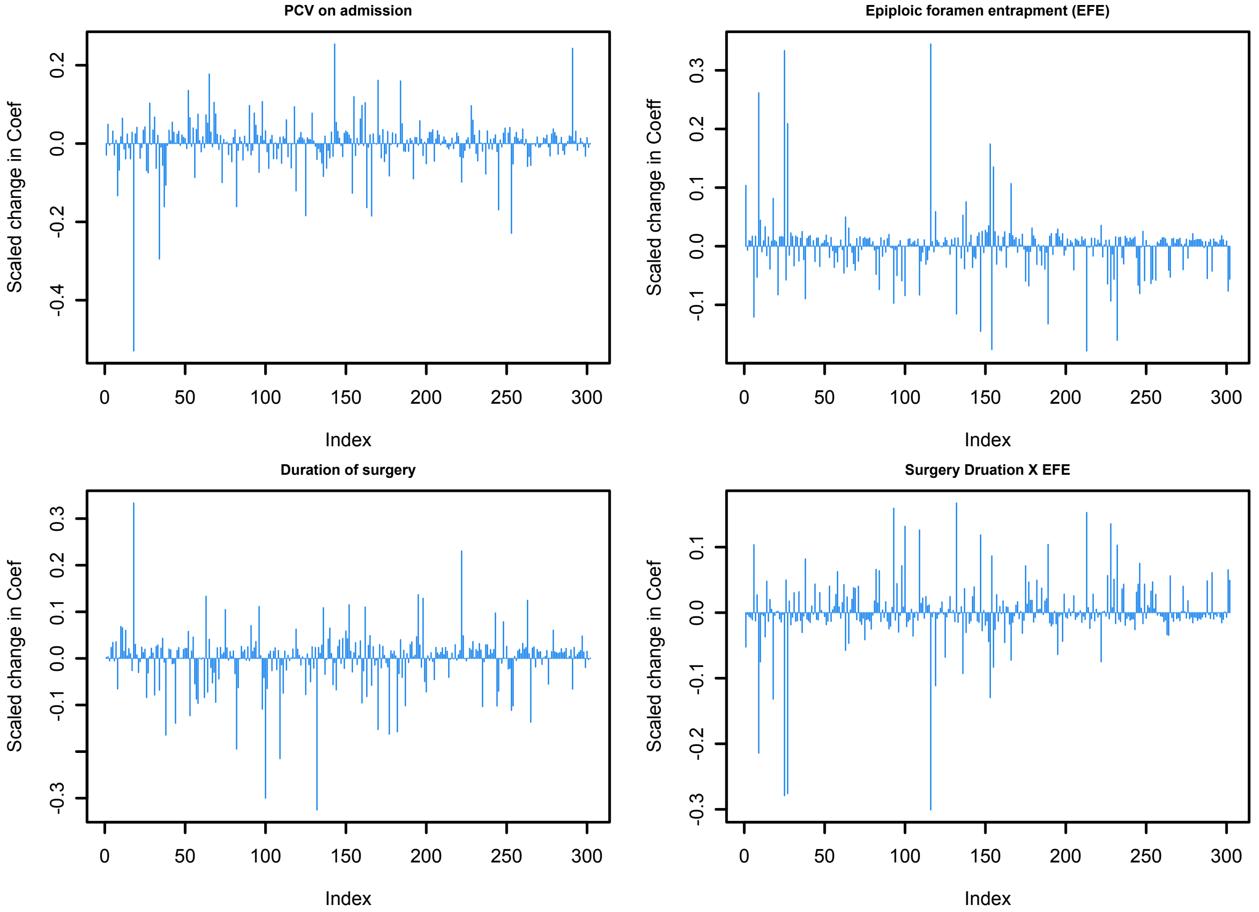

PCV on admission
Epiploic foramen entrapment (EFE)
Duration of surgery
Surgery Druation X EFE
Scaled change in Coef
Index
0
50
100
150
200
250
300
0.2
0.0
-0.2
-0.4
0.3
0.1
-0.1
-0.3
