# Supplementary figures and images for: Has intravenous lidocaine improved the outcome in horses following surgical management of small intestinal lesions in a UK hospital population?
Source: BMC Vet Res. 2016 Jul 27;12:157. doi: 10.1186/s12917-016-0784-7 (PMC4962447; doi:10.1186/s12917-016-0784-7)

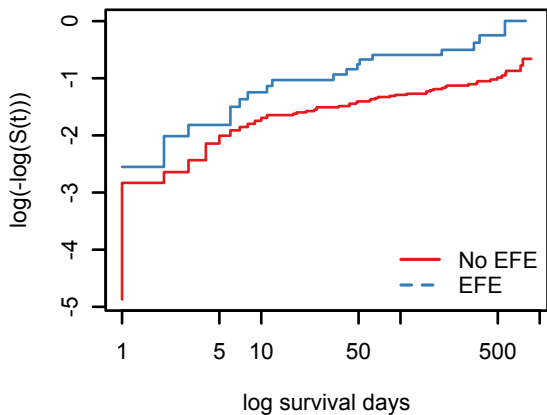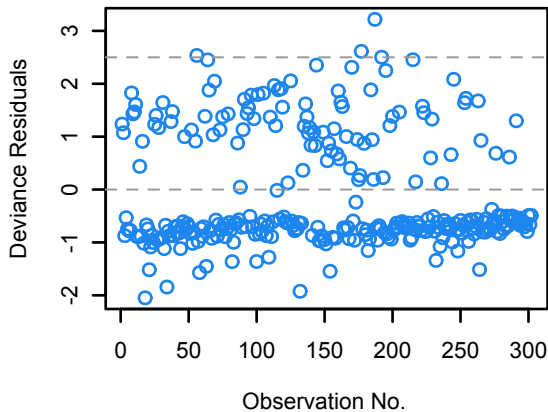

Supplement: Additional file 5: — Complementary log-log Kaplan-Meier survival curve for epiploic foramen entrapment (EFE) and a deviance residual plot. The complementary log-log Kaplan-Meier survival curve of EFE on a logarithmic scale (a) suggests that the proportional hazards assumption is satisfied for this variable (Therneau-Grambsch non-proportionality test p = 0.24). A deviance residual plot from the final multivariable Cox proportional hazards model (b) demonstrates five outlying data points (deviance residuals >2.5). (PDF 60 kb) [file 12917_2016_784_MOESM5_ESM.pdf]
